# Endogenous Galectin-1 Modulates Cell Biological Properties of Immortalized Retinal Pigment Epithelial Cells In Vitro

**DOI:** 10.3390/ijms241612635

**Published:** 2023-08-10

**Authors:** Caspar Liesenhoff, Simon Martin Paulus, Caroline Havertz, Arie Geerlof, Siegfried Priglinger, Claudia Sybille Priglinger, Andreas Ohlmann

**Affiliations:** 1 Department of Ophthalmology, University Hospital, LMU Munich, Mathildenstrasse 8, 80336 Munich, Germany; caspar.liesenhoff@med.uni-muenchen.de (C.L.); simon.paulus@med.uni-muenchen.de (S.M.P.); caroline.havertz@med.uni-muenchen.de (C.H.); siegfried.priglinger@med.uni-muenchen.de (S.P.); claudia.priglinger@med.uni-muenchen.de (C.S.P.); 2 Protein Expression and Purification Facility, Institute of Structural Biology, Helmholtz Center Munich for Environmental Health, 85764 Neuherberg, Germany; arie.geerlof@helmholtz-muenchen.de

**Keywords:** galectin-1, galectin-1 deficiency, galectin-1 knockout, retinal pigment epithelium cells, ARPE-19, cell proliferation, cell attachment, cell viability, epithelial–mesenchymal transition, compensatory gene expression

## Abstract

In the eye, an increase in galectin-1 is associated with various chorioretinal diseases, in which retinal pigment epithelium (RPE) cells play a crucial role in disease development and progression. Since little is known about the function of endogenous galectin-1 in these cells, we developed a galectin-1-deficient immortalized RPE cell line (ARPE-19-LGALS1^−/−^) using a sgRNA/Cas9 all-in-one expression vector and investigated its cell biological properties. Galectin-1 deficiency was confirmed by Western blot analysis and immunocytochemistry. Cell viability and proliferation were significantly decreased in ARPE-19-LGALS1^−/−^ cells when compared to wild-type controls. Further on, an increased attachment of galectin-1-deficient RPE cells was observed by cell adhesion assay when compared to control cells. The diminished viability and proliferation, as well as the enhanced adhesion of galectin-1-deficient ARPE-19 cells, could be blocked, at least in part, by the additional treatment with human recombinant galectin-1. In addition, a significantly reduced migration was detected in ARPE-19-LGALS1^−/−^ cells. In comparison to control cells, galectin-1-deficient RPE cells had enhanced expression of sm-α-actin and N-cadherin, whereas expression of E-cadherin showed no significant alteration. Finally, a compensatory expression of galectin-8 mRNA was observed in ARPE-19-LGALS1^−/−^ cells. In conclusion, in RPE cells, endogenous galectin-1 has crucial functions for various cell biological processes, including viability, proliferation, migration, adherence, and retaining the epithelial phenotype.

## 1. Introduction

Galectins are endogenous lectins that bind specifically to β-galactoside-binding sites via their conserved carbohydrate recognition domains [1]. Since β-galactosylation is a common glycosylation motive, many different intra- and extracellular proteins, as well as transmembrane proteins and receptors, have the potential to interact with various galectins [2]. However, besides their binding to β-galactoside moieties, galectins can also undergo non-carbohydrate-dependent protein–protein interactions [1,3].

Galectin-1 contains one carbohydrate recognition domain and forms homodimers with two galactoside-binding sites via sandwich binding of their two anti-parallel β-sheets [4]. Untypically for an extracellular protein, it is translated in the cytosol and secreted via a non-classical vesicle-mediated exocytosis, omitting the endoplasmic reticulum and Golgi apparatus [5]. In the cell, galectin-1 is located in the cytoplasm and in the nucleus, where it can interact with various proteins to modify signaling pathways, apoptosis, actin polymerization, or preRNA processing [4,6]. In the extracellular space, it is commonly located in the extracellular matrix or at the extracellular side of the plasma membrane, where it binds to glycosylated membrane proteins or receptors to modify their function [4]. For instance, by forming an extracellular lattice of membrane proteins or receptors, galectin-1 can modify their efficiency of endocytosis, which in turn can modulate the intracellular signal intensity of their respective downstream pathways [6,7]. Overall, in mammals, due to its various intra- and extracellular functions, galectin-1 is involved in physiological and pathological processes including tumor development and progression, immune and inflammatory response, diabetes mellitus and obesity, neuronal regeneration, and tissue fibrosis [8,9,10,11].

In the posterior eye segment, the retinal pigment epithelium is essential for the functional maintenance of photoreceptors and the choriocapillaris while also playing a crucial role in the pathology of several chorioretinal diseases. Intriguingly, in various pathologies of the chorioretinal interface, a role for galectin-1 as a potential modifier for disease progression is discussed. For instance, in the serum of patients with autoimmune uveitis, increased levels of α-galectin-1 antibodies were observed, which positively correlated with the severity of inflammation [12]. In line with these clinical observations, in experimental studies, an anti-inflammatory effect of galectin-1 was observed in a rodent model of endotoxin-induced uveitis [13]. Further on, in the vitreous bodies of patients suffering from severe proliferative diabetic retinopathy, significantly increased levels of galectin-1 were detected [14]. In vitro studies on immortalized RPE cells indicate that their incubation with high-glucose cell culture medium leads to enhanced expression of galectin-1 and reduced viability of the cells, an effect that could be reversed by the galectin-1 inhibitor OTX008 [15]. Moreover, in an experimental mouse model for age-related macular degeneration, the expression of galectin-1 was increased, while its lack substantially reduced the formation of choroidal neovascularization, a vision-threatening progression of the disease in humans [16]. In addition, in the preretinal membranes of patients suffering from proliferative vitreoretinopathy, a severe complication of retinal detachment, a strong signal for galectin-1 was detected [17]. In summary, all these studies indicate that galectin-1 could play a critical role in proliferative chorioretinal diseases. 

Although the pathogenesis of diseases of the chorioretinal interface is quite different, RPE cells commonly play a crucial role in their development and progression. However, only little is known about the specific function of galectin-1 in RPE cells under regenerative and pathological conditions. To further elucidate the role of galectin-1 in RPE cells, we developed a galectin-1-deficient immortalized RPE cell line and analyzed the effects of galectin-1 deficiency on several cell biological properties such as proliferation, migration, cell adhesion, epithelial-to-mesenchymal transition, and compensatory galectin expression in vitro. 

## 2. Results

### 2.1. Development of Galectin-1-Deficient ARPE-19 Cells

Since the biological properties of ARPE-19 cells depend on various factors such as cell culture medium and its supplementation or differentiation, the expression pattern of typical RPE genes was analyzed by real-time rt-PCR and compared to that of cultured human RPE cells after the first passage [18]. 

As reported previously by others, the expression of genes of the visual cycle (*STRA6*, *RPR65*) and RPE pigmentation (*TYR*) was very low in undifferentiated ARPE-19 cells under growing conditions when compared to cultured human RPE cells (Table 1) [19]. Further on, in ARPE-19 cells, the expression pattern for transport proteins was ambivalent since the mRNA level for monocarboxylate transporter 3 (*SLC16A8*) was suppressed by approximately 60%, whereas that of bestrophin-1 (*BEST1*) was only reduced by approximately 20% when compared to cultured human RPE cells (Table 1). In contrast, the markers for metabolism (ubiquinol-cytochrome c reductase core protein 2, *UQCRC2*), phagocytosis (integrin-αV, *ITGAV*; MER proto-oncogene, *MERTK*), cytoskeleton (cytokeratin 8 and 18, *KRT8*, *KRT18*), and secretion (pigment epithelium-derived factor, *SERPINF1*) were only moderately or slightly altered in ARPE-19 cells when compared to cultured human RPE cells (Table 1). 

For the development of galectin-1-deficient ARPE-19 cells, the all-in-one sgRNA pCRISPR plasmid against human LGALS1 (pCRISPR-LGALS1) was used. After transfection, the cells express the Cas9 protein and a specific guide RNA, which assemble into a functional and highly specific endonuclease targeting LGALS1. In addition, the vector expresses a green fluorescent protein (GFP) and a puromycin resistance gene for the selection of transfected cells. 

**Figure 1:**
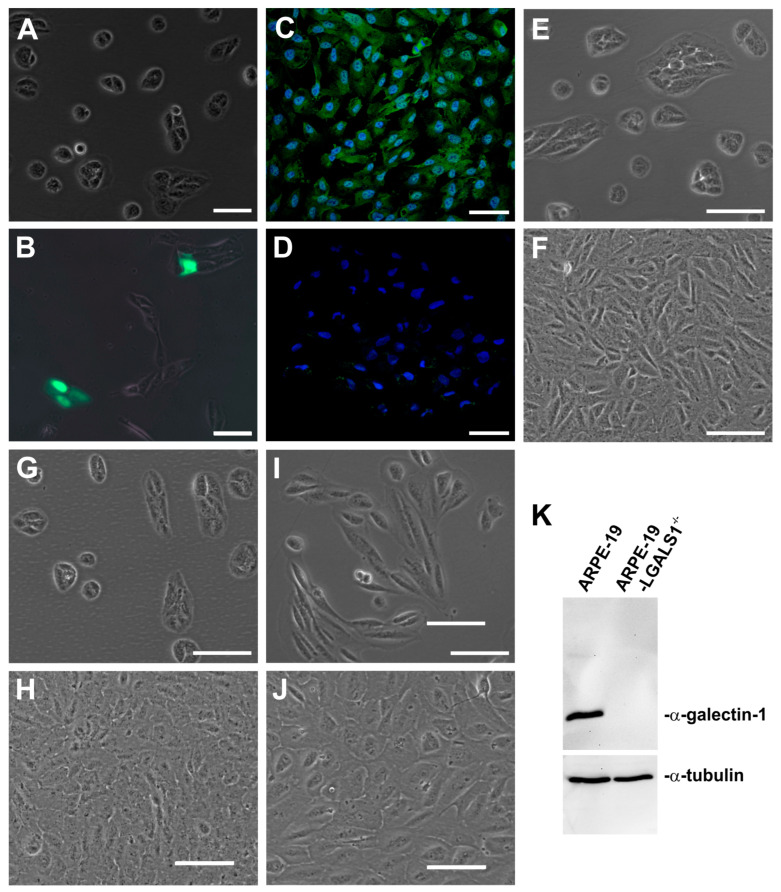
Development of galectin-1-deficient ARPE-19 cells. (**A**,**B**) Merged phase contrast and fluorescent imaging for GFP expression of ARPE-19 cells without (**A**) and with pCRISPR-LGALS1 transfection (**B**). (**C**,**D**) Immunostaining for galectin-1 in ARPE-19 (**C**) and ARPE-19-LGLAS1^−/−^ cells (**D**) after FACS and single-cell cultivation. (**E**–**J**) Phase contrast image of growing (**E**,**G,I**) and confluent (**F**,**H**,**J**) ARPE-19 (**E**,**F**), ARPE-19/FACS (**G**,**H**), and ARPE-19-LGLAS1^−/−^ cells (**I**,**J**). (**K**) Western blot analysis for galectin-1 in ARPE-19 and ARPE-19-LGLAS1^−/−^ cells. Magnification bar in (**A**,**B**): 100 µm; in (**C**,**D**): 50 µm; and in (**E**–**J**): 100 µm; blue, Hoechst staining.

Following transfection of ARPE-19 cells with the pCRISPR-LGALS1 vector, an intense signal for GFP was observed in approximately 5 to 10% of all cells, indicating successful transduction of the vectors (Figure 1A,B). For further selection, fluorescence-activated cell sorting (FACS) was performed, followed by the cultivation of single cells to obtain genetically homogeneous LGALS1-deficient ARPE-19 cells. Several potential ARPE-19-LGALS1^−/−^ cell clones were tested by Western blot analysis. Negatively tested cell clones were used as additional controls for the native ARPE-19 cells and are referred to as ARPE-19/FACS cells. One out of eight pCRISPR-LGALS1-transfected ARPE-19 cell clones had a complete lack of galectin-1 expression (Figure 1K). In line with our Western blot analyses, by immunocytochemistry, a complete loss of galectin-1 expression could be observed in ARPE-19-LGALS1^−/−^ cells, whereas native ARPE-19 cells showed the typical staining for galectin-1 in the nucleus and cytoplasm (Figure 1C,D). Further on, by phase contrast microscopy, ARPE-19-LGALS1^−/−^ cells (Figure 1I,J) showed a flattened round-to-oval or an elongated, spindle-shaped phenotype when compared to the cobblestone morphology of native ARPE-19 and ARPE-19/FACS cells (Figure 1E–H). 

### 2.2. Lack of Galectin-1 Decreases Viability and Proliferation of Immortalized RPE Cells In Vitro 

To analyze if galectin-1 affects the viability of ARPE-19 cells, the intracellular amount of reduced NADH in native ARPE-19 and ARPE-19-LGALS1^−/−^ cells was measured using a WST-1 assay. 

**Figure 2:**
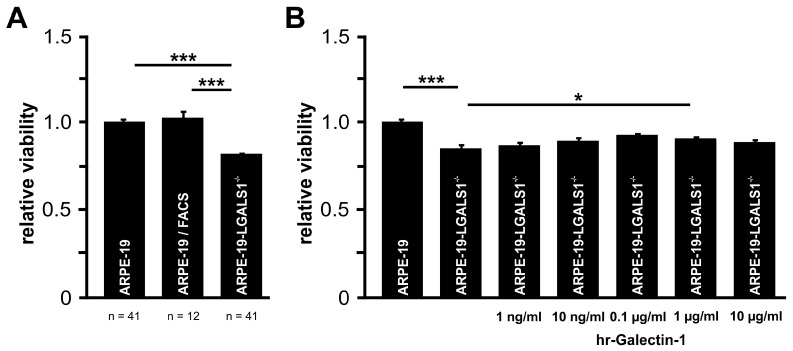
Lack of galectin-1 decreases viability in immortalized RPE cells in vitro. (**A**) WST-1 assay of ARPE-19, ARPE-19/FACS, and ARPE-19-LGALS1^−/−^ cells following incubation in cell culture medium without supplements for 72 h. Mean ± SEM; *** *p* < 0.001; n ≥ 12 of 3 or more independent experiments. (**B**) WST-1 assay of ARPE-19 and ARPE-19-LGALS1^−/−^ cells following incubation with indicated concentrations of rh-galectin-1 in cell culture medium without supplements for 72 h. Mean ± SEM; * *p* < 0.05, *** *p* < 0.001; n = 12 of 3 independent experiments.

After incubation of the cells in cell culture medium without supplements for 72 h, a negligible increase of less than 3% of WST-1 substrate turnover in ARPE-19/FACS cells was detected when compared to ARPE-19 cells (Figure 2A), suggesting that FACS sorting had no influence on their viability. However, in ARPE-19-LGALS1^−/−^ cells, a significant reduction in WST-1 substrate turnover to 81.9 ± 1.3% was observed when compared to native ARPE-19 (100.0 ± 1.9; *p* < 0.001) or ARPE-19/FACS cells (102.9 ± 2.9; *p* < 0.001; Figure 2A). 

To analyze if the reduced viability could be rescued, galectin-1-deficient ARPE-19 cells were treated with human recombinant (hr)-galectine-1. Following incubation of ARPE-19-LGALS1^−/−^ cells with various concentrations of hr-galectine-1 for 72 h, a dose-dependent moderate increase in WST-1 substrate turnover was observed (Figure 2B). The elevated viability was significantly enhanced following an incubation with 1 µg/mL hr-galectin-1 (90.6 ± 0.8, *p* = 0.021) when compared to galectin-1-deficient controls (85.9 ± 0.9), suggesting that the decreased viability of ARPE-19-LGALS1^−/−^ cells could be rescued by exogenous galectin-1. 

Since galectin-1 is known to modify cell proliferation in several cell types, we investigated the potential proliferative effects of galectin-1 on ARPE-19 cells in vitro.

**Figure 3:**
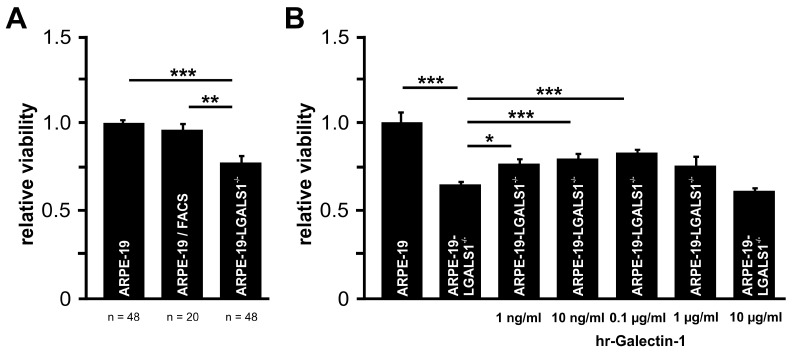
Lack of galectin-1 decreases proliferation in immortalized RPE cells in vitro. (**A**) BrdU ELISA of ARPE-19, ARPE-19/FACS, and ARPE-19-LGALS1^−/−^ cells following incubation in cell culture medium without supplements for 72 h. Mean ± SEM; ** *p* < 0.01, *** *p* < 0.001; n ≥ 20 of 4 or more independent experiments. (**B**) BrdU ELISA of ARPE-19 and ARPE-19-LGALS1^−/−^ cells following incubation with indicated concentrations of rh-galectin-1 in cell culture medium without supplements for 72 h. Mean ± SEM; * *p* < 0.05, *** *p* < 0.001; n ≥ 12 of 3 independent experiments.

After incubation of the cells in cell culture medium without supplements for 72 h, a marginal decrease in cell proliferation was observed in ARPE-19/FACS cells (96.3 ± 3.1%) when compared to wildtype controls, strongly suggesting that FACS sorting had no influence on ARPE-19 cell proliferation. In contrast, a significantly decreased proliferation of ARPE-19-LGALS1^−/−^ cells (78.3 ± 3.3%) was detected when compared to ARPE-19 (100.0 ± 1.5%; *p* < 0.001) or ARPE-19/FACS cells (96.3 ± 3.1%; *p* = 0.004; Figure 3A).

To investigate if the reduced proliferation could be reversed, ARPE-19-LGALS1^−/−^ cells were supplemented with various concentrations of hr-galectin-1. After incubation for 72 h, a dose-dependent increase in ARPE-19-LGALS1^−/−^ cell proliferation was observed, which was most pronounced and highly significant following incubation with 100 ng/mL hr-galectin-1 (83.6 ± 2.3%; *p* < 0.001) when compared to untreated galectin-1-deficient ARPE-19 cells (65.8 ± 0.8%; Figure 3B).

Overall, our data strongly suggest that galectin-1 promotes cell viability and proliferation of immortalized RPE cells in vitro, an effect that could be rescued by the addition of exogenous galectin-1. 

### 2.3. Lack of Galectin-1 Reduces Cell Migration of Immortalized RPE Cells on Plastic In Vitro

As migration is substantial not only for regeneration but also for pathological processes in RPE cells, we analyzed whether endogenous galectin-1 could affect cell migration of immortalized RPE cells in vitro by scratch migration wound healing assay.

**Figure 4:**
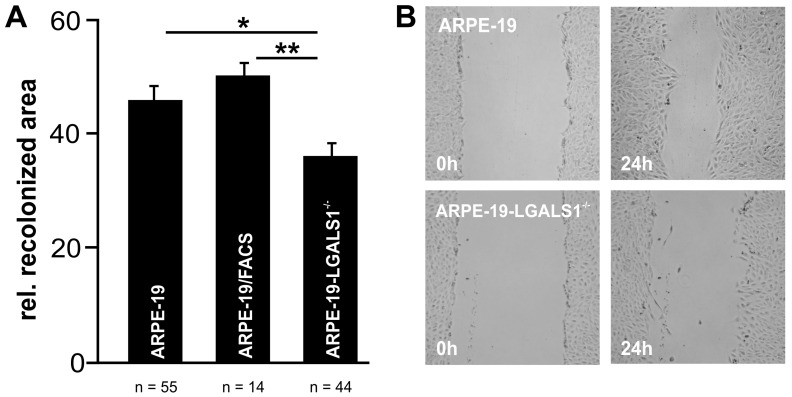
Lack of galectin-1 reduces cell migration of ARPE-19 cells on plastic in vitro. Representative images (**B**) and quantification (**A**) of scratch migration assays of ARPE-19, ARPE-19/FACS, and ARPE-19-LGALS1^−/−^ cells following incubation in cell culture medium without supplements for 24 h. (**A**) For quantification, the recolonized area after 24 h was calculated and plotted as the relative recolonized area. (**B**) The images on the left show the wounded area immediately after scratching (0 h), and the images on the right show the same region after incubation for 24 h. Mean ± SEM; * *p* < 0.05, ** *p* < 0.01; n ≥ 14 of at least 3 independent experiments.

Following incubation in cell culture medium without supplements for 24 h, 46.0 ± 2.4% and 50.4 ± 2.1% of the previously scratched area were covered with native ARPE-19 and ARPE-19/FACS cells, respectively, while the recolonized area of ARPE-19-LGALS1^−/−^ cells was 36.3 ± 2.5% (Figure 4A,B), an effect that was statistically significant for both control groups (ARPE-19, *p* = 0.014; ARPE-19/FACS, *p* = 0.003) and suggests a role of galectin-1 in the migration of immortalized RPE cells in vitro. 

### 2.4. Lack of Galectin-1 Enhances Cell Attachment in Immortalized RPE Cells In Vitro

Since cell attachment correlates with cell motility, which in turn is essential for tissue regeneration, we wondered if endogenous galectin-1 modifies cell adhesion in ARPE-19 cells.

**Figure 5:**
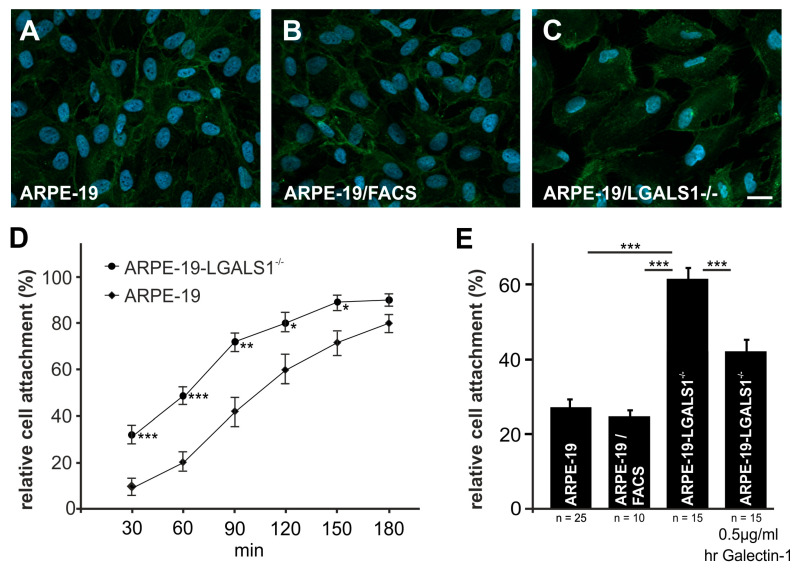
Endogenous galectin-1 decreases cell attachment in ARPE-19 cells in vitro. (**A**–**C**) Immunofluorescent staining against integrin-β1 of ARPE-19 (**A**), ARPE-19/FACS (**B**), and ARPE-19-LGALS1^−/−^ cells (**C**). Magnification bar in (**A**–**C**): 20 µm. (**D**) Cell adhesion analysis of ARPE-19 and ARPE-19-LGALS1^−/−^ cells following incubation in cell culture medium without supplements. The number of adherent cells was quantified 30, 60, 90, 120, 150, and 180 min after seeding. Mean ± SEM; n = 12 for each group of 3 independent experiments; * *p* < 0.05, ** *p* < 0.01, *** *p* < 0.001. (**E**) Cell adhesion of ARPE-19, ARPE-19/FACS, and ARPE-19-LGALS1^−/−^ cells with or without additional incubation with 0.5 µg/mL hr-galectin-1 and quantification of attached cells after 90 min. Mean ± SEM; n ≥ 10 for each group of at least 3 independent experiments; *** *p* < 0.001.

By cell adhesion assay, 30 min after seeding, significantly more ARPE-19-LGALS1^−/−^ cells (32 ± 4%, *p* < 0.001) were attached to the bottom of the cell culture dish when compared to native ARPE-19 cells (9 ± 8%, Figure 5D). Following prolonged incubation, the number of adherent cells further increased in both groups but was significantly higher in ARPE-19-LGALS1^−/−^ cells up to 150 min after seeding, strongly suggesting that galectin-1 decreases the adherence of immortalized ARPE-19 cells. 

Further on, for the rescue of increased attachment in galectin-1-deficient cells, ARPE-19-LGALS1^−/−^ cells were treated with 0.5 µg/mL hr-galectin-1. After incubation for 90 min, only 26 ± 2% and 25 ± 1% of wild-type ARPE-19 and ARPE-19/FACS cells, respectively, were attached to the bottom of the cell culture dish, whereas approximately 62 ± 3% of galectin-1-deficient cells were adherent (*p* < 0.001 for both controls). In contrast, following incubation with 0.5 µg/mL recombinant galectin-1, the number of attached galectin-1-deficient cells decreased by 17% when compared to untreated ARPE-19-LGALS1^−/−^ cells (45 ± 3%; *p* < 0.001; Figure 5E). Overall, our results strongly suggest that the enhanced adherence of galectin-1-deficient ARPE-19 cells is specifically caused by the lack of galectin-1 expression. 

Since integrin-β1 is known to mediate cell adhesion and is a potential binding partner for galectin-1, we investigated its expression by immunofluorescent staining. In ARPE-19, ARPE-19/FACS, and ARPE-19-LGALS1^−/−^ cells, a specific staining for integrin-β1 was detected at the cell membrane of all groups (Figure 5A–C). No obvious expression differences were observed between the groups, suggesting that integrin-β1 is expressed specifically and homogeneously at the cell membrane of galectin-1-deficient and control ARPE-19 cells.

### 2.5. Lack of Galectin-1 Promotes Epithelial-to-Mesenchymal Transition of Immortalized RPE Cells In Vitro

Since we observed an elongated, spindle-shaped phenotype in ARPE-19-LGALS1^−/−^ cells, we analyzed if the lack of galectin-1 leads to an epithelial-to-mesenchymal transition. 

**Figure 6:**
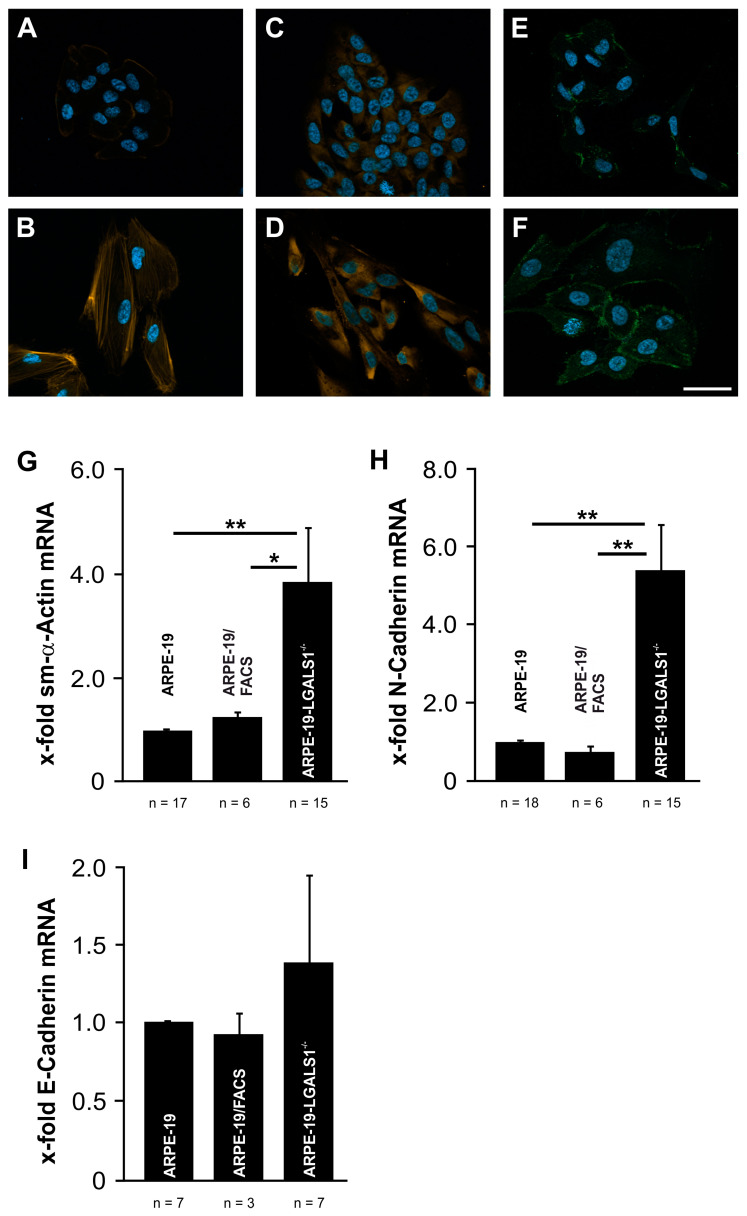
Endogenous galectin-1 expression is required for maintenance of the epithelial phenotype of immortalized RPE cells. Phalloidin (**A**,**B**, orange) as well as immunofluorescent staining for sm-α-actin (**C**,**D**, orange) and N-cadherin (**E**,**F**, green) of ARPE-19-LGALS1^−/−^ (**B**,**D**,**F**) and ARPE-19 cells (**A**,**C**,**E**). Magnification bars, 40 µm; blue, Hoechst staining. Real-time rt-PCR for sm-α-actin (**G**), N-cadherin (**H**), and E-cadherin (**I**) mRNA expression in ARPE-19-LGALS1^−/−^, ARPE19/FACS, and ARPE-19 cells. Mean ± SEM of more than 3 independent experiments; * *p* < 0.05, ** *p* < 0.01.

By phalloidin staining, a marker for stress fiber formation, only a weak signal was detected at the cell membrane of ARPE-19 cells (Figure 6A). In contrast, in galectin-1-deficient RPE cells, phalloidin-stained bundles spanning across the cytoplasm of the flattened and spindle-shaped cells were observed (Figure 6B). Further on, in ARPE-19-LGALS1^−/−^ cells, the immunohistochemical staining for smooth muscle (sm)-α-actin (Figure 6D) and N-cadherin (Figure 6F) was distinctly more intense when compared with wild-type ARPE-19 cells (Figure 6C,E).

To further confirm our observations, the mRNA levels for sm-α-actin, E-cadherin, and N-cadherin were analyzed. No significant difference in the mRNA expression of E-cadherin, a marker for the maintenance of an epithelial phenotype, was observed between ARPE-19-LGALS1^−/−^, ARPE-19/FACS, and ARPE-19 cells by real-time rt-PCR (Figure 6I). In contrast, in galectin-1-deficient ARPE-19 cells, a substantial increase in sm-α-actin mRNA was detected (3.47-fold ± 0.82; Figure 6G) when compared with ARPE-19/FACS (1.25-fold ± 0.08; *p* = 0.043) or ARPE-19 cells (1.0-fold ± 0.01; *p* = 0.001). In line with this, an enhanced expression of N-cadherin mRNA of 5.4-fold ± 1.14 was observed in ARPE-19-LGALS1^−/−^ cells in comparison to the ARPR-19/FACS (0.76-fold ± 0.13; *p* = 0.003) and ARPE-19 controls (1.0-fold ± 0.02; *p* = 0.005; Figure 6H). Overall, our data strongly indicate that endogenous expression of galectin-1 is essential to maintaining the epithelial phenotype of immortalized RPE cells in vitro.

### 2.6. Lack of Galectin-1 Induces Compensatory Expression of Galectin-8 in Immortalized RPE Cells In Vitro

To investigate whether the lack of endogenous galectin-1 leads to compensatory expression of other relevant galectins in RPE cells, the mRNA expression of galectin-1, -3, and -8 was analyzed in LGALS1-deficient ARPE-19 cells. 

**Figure 7:**
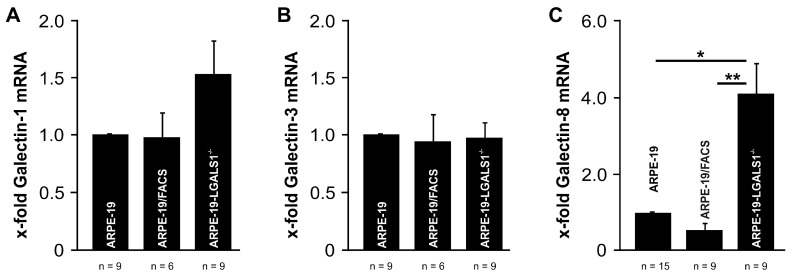
The lack of endogenous galectin-1 induces the expression of galectin-8 mRNA in immortalized RPE cells. Real-time rt-PCR for galectin-1 (**A**), -3 (**B**), and -8 (**C**) mRNA expression in ARPE-19-LGALS1^−/−^, ARPE-19/FACS, and ARPE-19 cells. Mean ± SEM of more than 6 independent experiments; * *p* < 0.05, ** *p* < 0.01.

In ARPE-19-LGALS1^−/−^ cells, only a slight increase in galectin-1 mRNA of approximately 50% and no alterations in galectin-3 mRNA level were observed when compared to ARPE-19/FACS or native ARPE-19 cells (Figure 7A,B). In contrast, for galectin-8, an enhanced expression of 4.1-fold ± 0.81 was detected in ARPE-19-LGALS1^−/−^ cells when compared with the ARPE-19/FACS (0.6-fold ± 0.127; *p* = 0.007) and wild-type ARPE-19 controls (1.0-fold ± 0.035; *p* = 0.034; Figure 7C), strongly indicating that the lack of galectin-1 leads to a compensatory enhanced expression of this galectin. 

## 3. Discussion

We conclude that galectin-1 is essential for the cell’s biological homeostasis and the maintenance of the epithelial phenotype of immortalized human RPE cells in vitro. Our conclusions rest upon (1) the observation that the lack of galectin-1 reduces cell proliferation and viability in immortalized RPE cells, (2) the finding that galectin-1 deficiency enhances adhesion and reduces migration of RPE cells, (3) the results that the lack of galectin-1 leads to a more mesenchymal phenotype and to an enhanced expression of sm-α-actin and N-cadherin in immortalized RPE cells, and finally (4) the observation that, in galectin-1-deficient ARPE-19 cells, the expression of galectin-8 is enhanced but not that of galectin-3. 

Galectin-1 is found extra- and intracellularly, where it can modulate a variety of processes via binding to specific proteins. For instance, extracellular galectin-1 can bind to specific membrane proteins, induce their clustering at the outer cell membrane, and thereby modify the function or signaling intensity of the bond proteins or receptors, respectively [7,20]. Further on, in the cell, galectin-1 can interact with proteins in the cytosol and the nucleus and thereby influence intracellular signaling, pre-mRNA processing in the nucleus, or the transcription of specific target genes [6,21]. Since galectin-1 can mediate its properties via intra- as well as extracellular interactions, we used the cell culture model of galectin-1-deficient immortalized RPE cells to analyze the role of galectin-1 under physiological conditions. For the characterization of the used ARPE-19 cells, the expression of several RPE-specific genes was detected and compared to that of cultured human RPE cells [18]. The expression of genes of the visual cycle and RPE pigmentation was very low in undifferentiated ARPE-19 cells under growing conditions, which is in line with the observations of other groups [19]. Since, except for these markers, our ARPE-19 cells have a comparable expression pattern to human cultured RPE cells, we used these cells to analyze the effects of endogenous galectin-1 on proliferating RPE cells in vitro. For the generation of ARPE-19-LGALS1^−/−^ cells, we used an all-in-one pCRISPR vector expressing the Cas9 protein, the specific gRNA for *LGALS1*, and a green fluorescent protein (GFP). The GFP expression of successfully transfected ARPE-19 cells enabled us to establish a clone-specific cell line following FACS analysis. It is known that the CRISPR/Cas9 technology has the limitation of unspecific DNA binding, which in turn could lead to adverse mutations in the genome and hence to a modified expression of the affected genes. By immunocytochemistry and Western blot analysis, we could clearly demonstrate that our ARPE-19-LGALS1^−/−^ cells have a complete lack of galectin-1 expression. In addition, by incubation with hr-galectin-1, we could attenuate the increased adherence of ARPE-19-LGALS1^−/−^ cells. In line with this, the reduced viability and proliferation of galectin-1-deficient ARPE-19 cells could be rescued, at least in part, by the additional treatment with hr-galectin-1 as well. These effects of hr-galectin-1 strongly indicate that the enhanced attachment as well as the diminished viability and proliferation of ARPE-19-LGALS1^−/−^ cells can be specifically attributed to the lack of galectin-1 expression. Further on, the mRNA expression of galectin-8 was significantly increased in ARPE-19-LGALS1^−/−^ cells, while those of galectin-1 and -3 were not changed. Since in several in vitro and in vivo knockout models an enhanced compensatory expression of closely related genes is reported, it is tempting to speculate if the lack of galectin-1 protein in RPE cells leads to an increased expression of galectin-8. Even though we cannot rule out completely that the effects observed in our galectin-1-deficient ARPE-19 cells are caused by unspecific CRISPR/Cas9 activity, there is strong evidence that the observed effects in our ARPE-19-LGALS1^−/−^ cells are specifically mediated by the deletion of the galectin-1 gene. 

By light microscopy, we observed a spindle-shaped, less epithelial phenotype of galectin-1-deficient ARPE-19 cells, which showed longitudinal stress fibers as well as an increased expression of N-cadherine and sm-α-actin. However, in several cell culture and animal studies, elevated levels of galectin-1 were associated with an epithelial–mesenchymal transition (EMT) of the cells investigated [16,22,23]. In line with this, in a mouse model of age-related macular degeneration, increased levels of galectin-1 enhanced the EMT of RPE cells and subretinal fibrosis via activation of the TGF-β pathway, whereas its knockdown blocked both effects [16]. However, an EMT is promoted by various pathways, including TGF-β, WNT, or Hedgehog signaling [24]. Intriguingly, for all these pathways, a modulating role of galectin-1 has been reported depending on the cell model and glycosylation of their transmembrane proteins [25,26,27]. Recent studies demonstrate that galectin-1 can not only enhance the signaling of specific pathways but also reduce the downstream activity of specific receptors via binding [28]. Since the cellular effects of galectin-1 depend on various circumstances, it is tempting to speculate if the lack of galectin-1 expression in immortalized RPE cells could promote dysbalanced signaling towards an EMT. 

In human immortalized ARPE-19 cells with a lack of galectin-1 expression, we observed reduced cell viability and proliferation when compared with the wild-type controls. The effects of galectin-1 on cell proliferation are complex and have therefore been extensively investigated in several normal or pathological cell lines from various tissues in humans or animals [4]. Depending on the cell line investigated, its intra- or extracellular location, the glycosylation pattern of proteins, and other factors, galectin-1 can promote or inhibit cell proliferation [4]. In order to clarify the role of galectin-1 on RPE cell proliferation under physiological conditions, we used our ARPE-19-LGALS1^−/−^ cells suffering from a complete lack of intra- and extracellular galectin-1. Even though we cannot rule out that galectin-1 treatment or specific pathological conditions may change galectin-1 function on RPE cell proliferation, we have strong evidence that galectin-1 in normal concentrations and conditions in RPE cells promotes their proliferation.

By mRNA analysis, we found a marked increase in galectin-8 expression in ARPE-19-LGALS1^−/−^ cells but no alteration in galectin-3 mRNA. In line with our observations, in several knockout models, upregulation of paralogues or genes of the same family has been reported to compensate for the lack of the specific gene [29]. Therefore, it is tempting to speculate that the increased expression of galectin-8 mRNA in ARPE-19-LGALS1^−/−^ cells could compensate, at least in part, for the lack of galectin-1. 

In RPE cells, the transmembrane protein integrin β1 is involved in mediating cell migration and attachment to the extracellular matrix and has the distinct potential to interact with galectin-1 [20,30,31,32]. In ARPE-19-LGALS1^−/−^ cells, we found a significant reduction in cell migration, which is in line with our previous study on RPE cells with a siRNA-mediated galectin-1 knockdown [33]. Further on, by cell adhesion assay, we observed an increased attachment of galectin-1-deficient ARPE-19 cells when compared with the wild-type controls. In previous studies, a decreased attachment of human RPE and human smooth muscle cells following treatment with recombinant galectin-1 in a dose-dependent manner was reported, which is in line with our results [34,35]. Since integrin β1 is involved in RPE cell migration and attachment and galectin-1 binding can modify its functions, it is most likely that the lack of galectin-1 expression could lead to an increased availability of integrin β1 on the cell membrane, which in turn could promote enhanced adherence and reduced migration of ARPE-19-LGALS1^−/−^ cells. 

Overall, we conclude that, in retinal pigment epithelial cells, galectin-1 has crucial functions for various cell biological processes, including viability, proliferation, migration, adherence, and retention of the epithelial phenotype, and thereby contributes to maintaining their homeostasis. 

## 4. Materials and Methods

### 4.1. Cell Culture

ARPE-19 cells (ATTC, Manassas, VA, USA) were maintained under standard culture conditions in an incubator (Thermo Fisher, Waltham, MA, USA) at 37 °C and 5% CO_2_. The cell culture medium (DMEM/HAM’s F12 medium, Bio&Sell, Nuernberg, Germany) was supplemented with 10% fetal calf serum, 50 μg/mL penicillin, and 50 μg/mL streptomycin (Merck, Darmstadt, Germany), and was changed every other day to provide standardized cultivation conditions. ARPE-19 cells were split at a confluence of 75% using versene solution with 0.05% trypsin (both from Thermo Fisher). Unless otherwise indicated, before the experiments, ARPE-19 cells were starved in culture medium without supplements for 24 h. For rescue experiments, cells were incubated with human recombinant galectin-1, which was expressed and isolated as described previously [36]. For immunocytochemistry, ARPE-19 cells were seeded on glass coverslips. 

Human cultured RPE cells were isolated from the eyes of human donors as described previously [34,37]. Methods for securing human tissue were humane, included proper consent and approval, complied with the Declaration of Helsinki, and were approved by the local ethics committee. After isolation, human RPE cells were cultured in DMEM (Bio&Sell) supplemented with 20% FCS, 50 μg/mL penicillin, and 50 μg/mL streptomycin (Merck). Following subculturing, primary RPE cells were maintained in DMEM (Bio&Sell) supplemented with 10% FCS, 50 μg/mL penicillin, and 50 μg/mL streptomycin. The epithelial origin of the cells was confirmed by immunostaining using cytokeratin-8 antibodies (Merck). 

### 4.2. Transfection

To establish a galectin-1-deficient ARPE-19 cell line, the all-in-one sgRNA pCRISPR plasmid against human LGALS1 (pCRISPR-LGALS1; HCP210588-CG04-3-B-b, Genecopoeia, Rockville, MD, USA), which promotes the expression of the specific gRNA and the CAS9 protein in transfected cells, was used. For selection, an additional neomycin resistance cassette and the cDNA for GFP were included in the plasmid. 

Prior to transfection, ARPE-19 cells were plated to approximately 60% confluence in a 24-well plate (Sarstedt, Nuembrecht, Germany). For transfection, 1.5 μL of Lipofectamine 3000, 2 μL of P3000 reagent (both from Thermo Fisher), and 1 µg of pCRISPR-LGALS1 plasmid were dissolved in 100 µL of DMEM/HAMs-F12 cell culture medium, incubated for 15 min at room temperature, and subsequently added to the ARPE-19 cells. Following incubation at 37 °C for 2 h, the cells were cultured in DMEM/Ham’s-F12 cell culture medium containing 10% fetal calf serum for an additional 24 h. To check for successfully transformed GFP-positive cells, the inverted fluorescence microscopy Axio Observer 7 containing an Apotome 2 was used (Zeiss, Oberkochen, Germany).

### 4.3. Fluorescence-Activated Cell Sorting

For selection of potential galectin-1-deficient cells, single-cell sorting of the previously transformed cells was performed using a FACSAriaTM III Cell Sorter (BD Life Sciences, San Jose, CA, USA) in accordance with the manufacturer’s recommendations. Cells with a 1000-fold higher GFP signal than wild-type ARPE-19 cells were defined as positively transformed. Following sorting, each positive cell was plated into a single well of a 96-well plate (Sarstedt) in DMEM/HAM’s F12 cell culture medium containing 10 ng/mL epithelial growth factor, 20 ng/mL fibroblastic growth factor-2 (both from Biolegend, San Diego, CA, USA), 20% FCS, 50 μg/mL penicillin, and 50 μg/mL streptomycin. After 48 h, the cell culture medium was changed, and cell cultivation was continued under standard conditions as described previously. 

### 4.4. Cell Proliferation and Cell Viability

Cell proliferation of LGALS1-deficient ARPE-19 cells was investigated using the 5-bromo-2’-deoxyuridine (BrdU) ELISA (Merck) in accordance with the manufacturer’s recommendations. In brief, 4,000 cells per well were seeded into a 96-well plate and incubated for 24 h under standard conditions to ensure complete cell adherence. Following an additional incubation with BrdU labeling solution for 24 h, cells were fixed and incubated with anti-BrdU antibodies. Following colorimetric development, the amount of BrdU incorporation into the DNA was quantified by absorbance measurement at a wavelength of 450 nm and a reference at 690 nm on the SpectraMax 190 ELISA reader (Molecular Devices, San Jose, CA, USA).

To analyze cell viability, the water-soluble tetrazolium dye (WST-1, Merck) was used in accordance with the manufacturer’s instructions. In brief, 10,000 cells per well were plated into a 96-well plate. Following attachment, cells were cultured in cell culture medium without supplements for 72 h. After an additional incubation in WST-1-containing cell culture medium for up to 2 h, absorbance measurements at a wavelength of 450 nm and a reference at 690 nm were performed on a SpectraMax 190 ELISA reader.

### 4.5. Scratch Migration Assay 

For scratch migration assay, cells were seeded in a 6-well plate and cultured until full confluency. By using a 100 μL pipette tip, a wound area was scratched, followed by two washes with 1× PBS to remove cell debris. Immediately after wounding as well as 24 h thereafter, the scratch was documented using an inverted Axio Observer 7 (Zeiss, Oberkochen, Germany). For quantification, the area before incubation and after 24 h was measured using the ZEN software Blue edition 3.0 (Zeiss) and plotted as relative recolonized area. 

### 4.6. Cell Adhesion Assay 

To analyze cell adherence, 1000 cells/cm^2^ were seeded onto a 6-well plate. After 30, 60, 90, 120, and 150 min, the cells were documented using an inverted Axio Observer 7 with ZEN software Blue edition 3.0. For quantification, the number of adherent cells was calculated and plotted as relative number of total cells. 

### 4.7. Immunocytochemistry 

Following fixation with 4% paraformaldehyde for 10 min, cells were washed 3 times for 5 min with 0.1 M phosphate buffer (0.1 M Na_2_HPO_4_ × 2H_2_O, 0.1 M NaH_2_PO_4_ × H_2_O, pH 7.4) and blocked with 3% bovine serum albumin and 0.1% Triton X-100 in 0.1 M phosphate buffer for 30 min. After incubation at 4 °C overnight with 1:50 rabbit α-galectin-1 (Abcam, Cambridge, UK), 1:100 mouse α-smooth muscle-α-actin (Santa Cruz, Dallas, TX, USA), 1:100 mouse α-N-cadherin (Thermo Fisher), 1:100 mouse α-integrin-β1 (Merck), 1:100 mouse α-cytokeratin-8 (Merck), 1:100 phalloidin Alexa Fluor 555 (Thermo Fisher) in 0.3% bovine serum albumin, and 0.01% Triton X-100 in 0.1 M phosphate buffer, specimens were washed again 3 times for 10 min each with 0.1 M phosphate buffer and incubated with 1:1000 goat anti-rabbit Alexa Fluor 488 or goat anti-mouse Alexa Fluor 488 (both from Thermo Fisher) in 1:10 diluted blocking solution for 1 h at room temperature. After nuclear staining with Hoechst 33342 (Thermo Fisher), specimens were washed again 3 times with 0.1 M phosphate buffer and mounted with the ProLong glass antifade mounting medium (Thermo Fisher). Immunofluorescence staining was analyzed by an Axio Observer 7 fluorescence microscope with an Apotome 2 module (Zeiss) and documented using the ZEN software Blue edition 3.0 (Zeiss). 

### 4.8. Protein Preparation and Western Blot Analysis 

ARPE-19 cells were lysed in RIPA buffer containing protease and phosphatase inhibitors (Complete; Merck). Up to 15 µg of the total protein per sample was loaded onto a 12% SDS-PAGE and transferred onto a PVDF membrane (Roche, Mannheim, Germany) by semidry blotting following electrophoresis. After blocking with 3% skimmed milk powder in 1× PBST, membranes were incubated with 1:1000 rabbit α-galectin-1 (Abcam, Cambridge, England) in 1× PBS with 0.3% skimmed milk powder and 0.1% Tween 20 at 4 °C overnight. Following 3 washes with 1× PBS for 10 min each, the membranes were hybridized with 1:1000 alkaline phosphatase (AP)-conjugated goat α-rabbit antibody (DIANOVA, Hamburg, Germany) in 1× PBS with 0.3% skimmed milk powder and 0.1% Tween 20. As loading control, blots were incubated with 1:5000 goat α-tubulin antibodies (R&D System, Minneapolis, MN, USA) in 1× PBS with 0.3% skimmed milk powder and 0.1% Tween 20 overnight, followed by an additional hybridization with 1:1000 AP-conjugated donkey α-goat antibodies (Dianova, Hamburg, Germany) in 1× PBS with 0.3% skimmed milk powder and 0.1% Tween 20 for 1 h. For visualization, membranes were incubated with CDP-Star substrate in accordance with the manufacturer’s recommendations (CDP-Star, Thermo Fisher) and documented with an iBrightCL1000 Imaging System (Thermo Fischer).

### 4.9. RNA Isolation, cDNA Synthesis, and Real-Time rt-PCR

For the expression analyses, ARPE-19-LGALS1^−/−^ and ARPE-19 cells were homogenized in TriFast (VWR, Leuven, Belgium), and total RNA was isolated according to the manufacturer’s recommendations. The concentration of the RNA and the OD260/OD280 ratio were measured with a BioPhotometer (Eppendorf, Hamburg, Germany). Only total RNA with a 260/280 ratio between 1.8 and 2.0 was used for first-strand cDNA synthesis with the iScript gDNA CLR cDNA Synthesis Kit (Bio-Rad, Hercules, CA, USA) in accordance with the manufacturer’s instructions. Quantitative real-time rt-PCR analyses were performed on a Bio-Rad CFX real-time PCR Detection System using the 2× iTaq Universal SYBR Green SMX mix (both Bio-Rad) in accordance with the manufacturer’s protocol. PCR was performed in a final volume of 15 µL, consisting of 7.5 µL of 2× iTaq Univer SYBR Green SMX mix and 0.16 µL of primer mix (1 µM each, Thermo Fisher). After initial activation at 95 °C for 3 min, the temperature profile was 10 s denaturation at 95 °C and 1 min annealing and extension at 60 °C for 40 cycles. RNA that had not been reverse-transcribed served as a negative control for real-time rt-PCR. All PCR primers were designed to span exon–intron boundaries and were purchased from Thermo Fisher (Table 2). For relative quantification, the reference gene GNB2L was used. Results were analyzed using the Bio-Rad CFX manager (version 3.1).

### 4.10. Statistical Analysis

All calculations and statistical analyses were performed using Excel 365 version 16 (Microsoft, Redmond, WA, USA) and SPSS version 22 (IBM, Armonk, NY, USA). For data presentation, the mean and the standard error of the mean (SEM) were calculated and shown as indicated. For comparison of two groups of mean variables, a Student’s *t*-test was used, and for more than two groups, a 1-way ANOVA test was used. For data that met the assumption of homogeneity of variances, a least significant difference (LSD) post hoc test was performed, and for data not meeting the criteria, a Games–Howell post hoc test was performed. *p* values less than 0.05 were considered statistically significant. 

## Figures and Tables

**Table 1:** Expression pattern of ARPE-19 cells compared to cultured human RPE cells.

Function	Gene	Number	Human RPE Cells	ARPE-19 Cells
			Mean ± SEM	Mean ± SEM
Visual cycle	*STRA6*	n = 3	1.0 ± 0.095	0.016 ± 0.007
	*RPE65*	n = 6	1.0 ± 0.520	0.071 ± 0.049
Melanogenesis	*TYR*	n = 6	1.0 ± 0.242	0.140 ± 0.018
Transport	*BEST1*	n = 3	1.0 ± 0.524	0.831 ± 0.259
	*SLC16A8*	n = 6	1.0 ± 0.074	0.359 ± 0.109
Metabolism	*UQCRC2*	n = 6	1.0 ± 0.221	1.181 ± 0.238
Phagocytosis	*MERTK*	n = 3	1.0 ± 0.088	1.043 ± 0.191
	*ITGAV*	n = 3	1.0 ± 0.208	0.749 ± 0.100
Cytoskeleton	*KRT8*	n = 3	1.0 ± 0.039	0.786 ± 0.016
	*KRT18*	n = 6	1.0 ± 0.174	0.897 ± 0.191
Secretion	*SERPINF1*	n = 3	1.0 ± 0.276	0.800 ± 0.072

**Table 2:** Primers used for real-time rt-PCR.

Gene	Accession No.	Sequence	Product Size
*BEST*	NM_004183.3	5′-agctgctatatggcgagttctt-3′ 5′-gctgttgttcttccgtgagg-3′	90 bp
*E-cadherin*	NM_004360	5′-cccgggacaacgtttattac-3′ 5′-gctggctcaagtcaaagtcc-3′	71 bp
*GNB2L*	NM_006098	5′-ctacaatgatctttccctctaaatcc-3′ 5′-cctaaccgctactggctgtg-3′	72 bp
*ITGAV*	NM_002210.5	5′-gactcctgctacctctgtgc-3′ 5′-agaaacatccgggaagacgc-3′	133 bp
*KRT8*	NM_001256282.2	5′-aatgaatggggtgagctgga-3′ 5′-atggacatggtagaggcagg-3′	93 bp
*KRT18*	NM_000224.3	5′-acatccgggcccaatatgac-3′ 5′-tggtgctctcctcaatctgc-3′	87 bp
*LGALS1*	NM_002305	5′-cgccagcaacctgaatct-3′ 5′-caggttcagcacgaagctct-3′	88 bp
*LGALS3*	NM_002306	5′-cttctggacagccaagtgc-3′ 5′-aaaggcaggttataaggcacaa-3′	94 bp
*LGALS8*	NM_201544	5′-cggtaatcccgtttgttggc-3′ 5′-cacctggaatctgtctgcgt-3′	100 bp
*N-cadherin*	NM_001308176	5′-ggtggaggagaagaagaccag-3′ 5′-ggcatcaggctccacagt-3′	72 bp
*MERTK*	NM_006343.3	5′-aaattacagatccgcagccc-3′ 5′-taaggcttggcttcttccct-3′	119 bp
*RPE65*	NM_000329.2	5′-ccctcctgcacaagtttgac-3′ 5′-tcagtcattgcccgtacgta-3′	91 bp
*SERPINF1*	NM_002615.7	5′-cgtatccacaggccccag-3′ 5′-gttctggcagctgctgtg-3′	82 bp
*sm-α-actin*	NM_001613	5′-ctgaagtacccgatagaacatgg-3′ 5′-ttgtagaaagagtggtgccagat-3′	77 bp
*SLC16A8*	NM_013356.3	5′-caggagatgtgaggctggac-3′ 5′-gctgtgcgtgattttcctct-3′	111 bp
*STRA6*	NM_001142617.2	5′-gggacaagtttccgggagag-3′ 5′-ctggcccttctcctccaatc-3′	92 bp
*TYR*	NM_000372.5	5′-agtacatgggaggtcagcac-3′ 5′-tggctgttgtactcctccaa-3′	106 bp
*UQCRC2*	NM_003366.4	5′-cctgcggggtgatgttgata-3′ 5′-cagctacttcccaacgacga-3′	83 bp

## Data Availability

The data presented in this study are available in the article.
